# Outcome after prenatal diagnosis of fetal urinary tract abnormalities: A tertiary center experience

**DOI:** 10.4274/jtgga.2017.0132

**Published:** 2018-11-15

**Authors:** Ayşegül Özel, Ebru Alıcı Davutoğlu, Hakan Erenel, Mehmet Fatih Karslı, Sevim Özge Korkmaz, Rıza Madazlı

**Affiliations:** 1 Department of Obstetrics and Gynecology, Perinatology Unit, İstanbul University Cerrahpaşa Faculty of Medicine, İstanbul, Turkey

**Keywords:** Prenatal, fetal, urinary tract, pelviectasis

## Abstract

**Objective:**

With the widespread use of ultrasonography for fetal screening, the detection and management of congenital urinary tract abnormalities has become crucial. In this study, we aimed to describe the clinical approaches in patients with prenatally detected urinary tract abnormalities.

**Material and Methods:**

This study is a retrospective, single-center study performed at a perinatology unit of a university hospital, between 2010 and 2016. The outcomes of 124 patients who were prenatally diagnosed as having urinary tract abnormalities are reported. Variables included in the analysis were fetal sex, birth week and weight, persistency, and necessity surgery after birth for renal pelvic dilatation. Low-risk renal pelvic dilatation was determined as an anterior-posterior (AP) diameter of 4-7 mm at 16-28 weeks, 7-10 mm after 28 weeks, whereas high-risk dilatation was defined as AP measurements of ≥7 mm at 16-28 weeks, ≥10 mm after 28 weeks, respectively.

**Results:**

The majority of patients consisted of male fetuses with bilateral pelviectasis (62.9%, 20.2%, respectively). The mean age was 28.8±6.4 years. The mean gestational age at birth was 34.2±7.8 weeks. The mean birth weight was 2593±1253.3 g. The need for surgery was greater in high-risk patients than in low-risk patients (58.3% vs. 8.7%) (p<0.002).

**Conclusion:**

Patients with high-risk antenatal renal pelvic dilatation require surgical treatment after delivery. Close prenatal and postnatal follow-up is mandatory in specialized centers. Perinatologists, neonatologists, pediatricians and pediatric nephrologists, and radiologists should treat these children with a multidisciplinary approach.

## Introduction

With a prevalence of 0.1-2.3%, urinary tract abnormalities are the most frequent findings on prenatal ultrasound (US) (1). The detection of these conditions in utero has permitted their early management. Nevertheless, patients are worried about abnormal findings on fetal US. Parents become strongly concerned and interested in prognosis, need of surgery and associated risks for their unborn baby (2,3). Oligohydramnios, bladder outlet obstruction, renal cysts, extra renal pathologies, prematurity, and low birth weight are adverse prognostic factors for postnatal outcome. It is still a matter of debate as to which specific prognostic factors predict termination of pregnancy (4). Prenatal pelvic dilatation has prognostic value and some studies suggested that it was correlated with the postnatal need for surgical treatment (5,6).

Congenital urinary tract abnormalities may develop at the level of the kidney (e.g., dysplasia and hypoplasia), collecting system (e.g., hydronephrosis and megaureter), bladder (e.g., ureterocele and vesicoureteral reflux), or urethra [e.g., posterior urethral valves (PUV)]. There is a continuous advance in the understanding of the genetic basis, pathophysiology, and natural history of these abnormalities (7). Renal pelvic dilatation of the fetus may be a cue for urinary tract abnormalities, ranging from obstruction to vesicoureteric reflux. However, pelviectasis may also be a marker for aneuploidy with increased incidence in fetuses with trisomy 21 (8,9); isolated urinary tract abnormalities have a low association with karyotypic abnormalities. Severe renal pelvic dilatation is associated with postnatal pathology and often requires surgical treatment in childhood (10).

In this retrospective study, we investigated the clinical course of prenatally diagnosed fetuses with urinary tract abnormalities and the relationship between fetal pyelectasis and need for postnatal treatment.

## Material and Methods

The records of 124 patients with prenatally detected congenital urinary tract abnormalities who were examined at the perinatology unit of a university hospital in İstanbul between 2010 and 2016 were reviewed, retrospectively. Ethics Committee approval was received. Each fetus underwent a detailed sonographic examination for detecting fetal organ abnormalities. The presence of renal, ureteral, and bladder abnormalities and volume of amniotic fluid, date (weeks of pregnancy) of first diagnosis, fetal sex, prenatal invasive genetic tests, and birth week and weight were recorded. Registers of birth of 16 patients could not be accessed. Fetuses with renal pelvic dilatation were also evaluated. The fetuses were divided into two groups as low-risk and high-risk. Low-risk renal pelvic dilatation was defined as an anterior-posterior (AP) diameter (in the transverse plane) of 4-7 mm at 16-28 weeks, and 7-10 mm after 28 weeks, whereas high-risk dilatation was defined as AP measurements of ≥7 mm at 16-28 weeks, and ≥10 mm after 28 weeks (11). If renal pelviectasis were found bilaterally, the largest diameter dilatation was used to classify the patient. The presence of unilateral or bilateral dilatation was also recorded. We learned about postnatal persistency and need for surgery through telephone interviews with their parents.

### Statistical analysis

We used SPSS^®^ software, version 20.0 (IBM Corp. Released 2011. IBM SPSS Statistics for Windows, Version 20.0. Armonk, NY: IBM Corp) to analyze the collected data. Data are summarized as mean ± standard deviation or numeric (%) as appropriate.

## Results

Retrospective data of 124 patients were analyzed. The clinical characteristics of the patients are summarized in Table 1. There were higher proportions of males and patients with bilateral pelviectasis (62.9%, 20.2%, respectively). The mean age of the mothers was 28.8±6.4 years. The mean gestational age at birth was 34.2±7.8 weeks. The mean birth weight was 2593±1253.3 g.

There were 4 (3.2%) neonatal exitus; 2 of which had bilateral renal agenesia, one neonate had infantile polycystic kidney, and one neonate had PUV. There were 6 (4.8%) spontaneous abortus and intrauterine demise; 2 of which had infantile polycystic kidney, two had megacystisis, one had unilateral dysplastic kidney, and one had PUV. There were 16 (12.9%) terminations of pregnancy; 6 fetuses had megacystisis, 4 had infantile polycystic kidney, two had PUV (one of which had trisomy 18), one had renal agenesia, one had bladder extrophy, one was multicystic dysplastic, and one had hyperechoic kidney. The birth records of the 16 patients could not be accessed.

Of the 41 patients with renal pelvic dilatation, 25 (60.9%) were low-risk, and 16 (39.1%) were high-risk. Of the 35 cases for which follow-up data were available, postnatal persistency and requirement for surgery within the first year of life were evaluated (Table 2). Renal pelvic dilatation was persistent in 15 (65.2%) patients who were defined as low-risk. Renal pelvic dilatation was persistent in all patients who were defined as high-risk. The need for surgery was significantly greater in high-risk patients than in low-risk patients (58.3% vs. 8.7%) (p<0.002).

## Discussion

Congenital abnormalities of the kidney and urinary tract account for 30-50% of all fetal anomalies. They occur with a prevalence of 1 in 70-1000 live births as the most common prenatal diagnoses. In addition, these abnormalities are the most common reason for chronic kidney disease in childhood (12). There is a wide spectrum of fetal anomalies, ranging from mild unilateral pelvic dilatation to severe bilateral renal and urinary tract malformations (13). Postnatal management of infants with a history of antenatal pelviectasis remains controversial, especially with regards to fetal intervention, diagnostic criteria, postnatal recommendations, and therapeutic management (14). The parents of fetuses with prenatal pelviectasis may be more concerned that their children will need advanced investigation and treatment after delivery, and how their renal function will be in the future. They are less interested in the most accurate diagnosis, but they are interested in the prognosis.

Unilateral pelviectasis typically requires no specific interventions during the prenatal period beyond close serial imaging. Bilateral pelviectasis, on the other hand, can be present in the context of clinically significant urinary tract obstruction such as PUV or urethral atresia, as well as in non-obstructing entities such as prune-belly syndrome or high-grade vesicoureteral reflux (2).

There are variable identification and classification schemes for  the definition of hydronephrosis. An AP pelvic diameter of ≥4 mm at the first trimester is the most commonly used cut-off to indicate pelviectasis (15,16,17). Ouzounian et al. (18) showed that fetal pelvic dilatation of 8 mm provided the best combination of sensitivity and specificity, at 91% and 72%, respectively. A study showed that a third trimester AP pelvis diameter of ≥7 mm was the strongest ultrasound (US) criterion to predict postnatal kidney pathologies (19). In order to predict prognosis in this study, we used a grading system that was defined at a consensus meeting (11). Low-risk renal pelvic dilatation was defined as an AP diameter of 4-7 mm at 16-28 weeks, and 7-10 mm after 28 weeks, whereas high-risk dilatation was defined as AP measurements of ≥7 mm at 16-28 weeks, and ≥10 mm after 28 weeks. The postnatal surgery rate was 58.3% in fetuses with high-risk renal pelvic dilatation. All fetuses with high-risk were persistent in the postnatal period, but this was 65.2% among fetuses with low risk.

There are two previous reports examining the incidence of postnatal surgery associated with antenatal pelviectasis, and both showed similar results (5,15). Sairam et al. (15) and Wollenberg et al. (5), demonstrated that 34% and 36%, respectively, of the fetuses with AP diameters ≥10 mm on US examination needed surgery. Our results were similar to the first study by Grignon et al. (20), but they reported a higher rate of surgical treatment (60%) in fetuses with AP diameter ≥10 mm. Differences in criteria used to indicate surgery probably account for the differences in surgical treatment rates. John et al. (21) showed that fetuses with AP diameters ≥19 mm after 33 weeks’ gestation had a significant risk of postnatal surgery. They also reported a spontaneous recovery rate of 25% at three months after birth, including children with fetal hydronephrosis defined as AP diameter ≥4 mm until 33 weeks’ gestation and AP diameter ≥7 mm thereafter.

### Study limitations

There are potential limitations associated with the retrospective design in our study. In addition, the small number of cases is another limitation. Therefore, we could not use multivariate analysis to describe possible prognostic factors.

Our investigation showed that two-thirds (58%) of patients with high-risk antenatal renal pelvic dilatation required surgical treatment after delivery. Close prenatal and postnatal follow-up is mandatory in specialized centers. Perinatologists, neonatologists, pediatricians and pediatric nephrologists, and radiologists should have a multidisciplinary approach for these children. The ability to effectively determine fetuses with high-risk pelviectasis in the antenatal period would provide correct postnatal management. It is important for minimizing unnecessary parental anxiety and postnatal renal damage.

## Figures and Tables

**Table 1:**
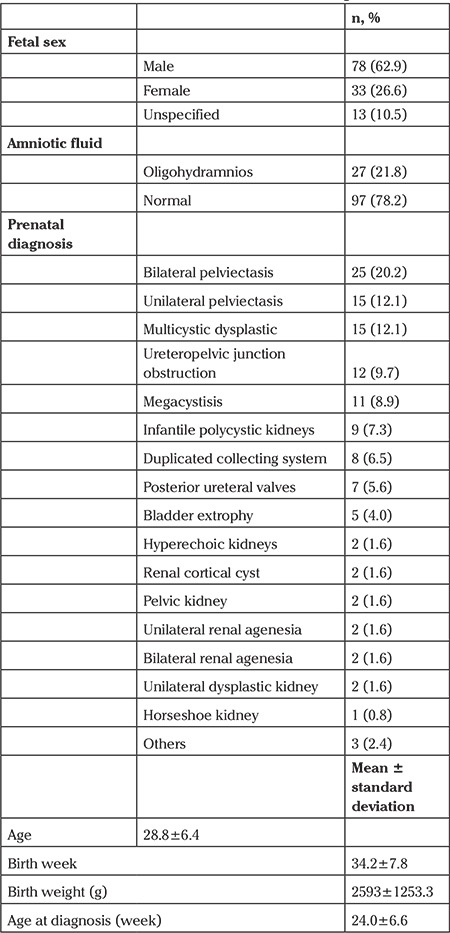
The main characteristics of the patients

**Table 2:**
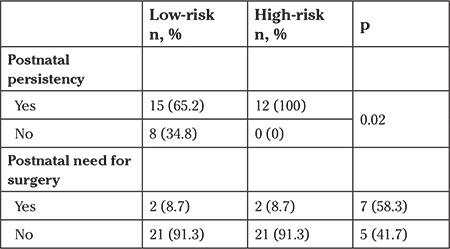
Postnatal persistency and surgery rates in the low and highrisk groups
